# Effects of Lipid Composition and Solution Conditions on the Mechanical Properties of Membrane Vesicles

**DOI:** 10.3390/membranes5010022

**Published:** 2015-01-20

**Authors:** Nobuhiko Kato, Akihiko Ishijima, Takehiko Inaba, Fumimasa Nomura, Shuichi Takeda, Kingo Takiguchi

**Affiliations:** 1 Division of Biological Science, Graduate School of Science, Nagoya University, Chikusa-ku, Nagoya 464-8602, Japan; E-Mails: k614899x@m2.aichi-c.ed.jp (N.K.); takeda.shuichi@f.mbox.nagoya-u.ac.jp (S.T.); 2 Institute of Multidisciplinary, Research for Advanced Materials, Tohoku University, 2-1-1, Katahira, Aoba-ku, Sendai 980-8577, Japan; E-Mail: ishijima@tagen.tohoku.ac.jp; 3 Lipid Biology Laboratory, RIKEN, 2-1 Hirosawa, Wako, Saitama 351-0198, Japan; E-Mail: takinaba@riken.jp; 4 Department of Biomedical Information, Division of Biosystems, Institute of Biomaterials and Bioengineering, Tokyo Medical and Dental University, 2-3-10 Kanda-Surugadai, Chiyoda, Tokyo 101-0062, Japan; E-Mail: nomura.bmi@tmd.ac.jp; 5 Structural Biology Research Center, Nagoya University, Chikusa-ku, Nagoya 464-8601, Japan

**Keywords:** membrane vesicles, giant unilamellar liposome, laser tweezers, mechanical processes, biomechanical phenomena, real-time imaging

## Abstract

The mechanical properties of cell-sized giant unilamellar liposomes were studied by manipulating polystyrene beads encapsulated within the liposomes using double-beam laser tweezers. Mechanical forces were applied to the liposomes from within by moving the beads away from each other, which caused the liposomes to elongate. Subsequently, a tubular membrane projection was generated in the tip at either end of the liposome, or the bead moved out from the laser trap. The force required for liposome transformation reached maximum strength just before formation of the projection or the moving out of the bead. By employing this manipulation system, we investigated the effects of membrane lipid compositions and environment solutions on the mechanical properties. With increasing content of acidic phospholipids, such as phosphatidylglycerol or phosphatidic acid, a larger strength of force was required for the liposome transformation. Liposomes prepared with a synthetic dimyristoylphosphatidylcholine, which has uniform hydrocarbon chains, were transformed easily compared with liposomes prepared using natural phosphatidylcholine. Surprisingly, bovine serum albumin or fetuin (soluble proteins that do not bind to membranes) decreased liposomal membrane rigidity, whereas the same concentration of sucrose showed no particular effect. These results show that the mechanical properties of liposomes depend on their lipid composition and environment.

## 1. Introduction

Liposomes, which are artificial membrane vesicles, are a useful model to study how the shape and morphology of lipid bilayer membranes are controlled by external factors, such as physical parameters and chemical or biological agents. Liposomes have been used to study the relationships between membrane morphology and phase separation that take place in the membrane or spontaneous curvature, which is attributable to the molecular shape of the constituent lipids or differences in the number of lipid molecules between its two leaflets [1,2,3]. The conditions of the solutions on both sides of the membrane are also important in the determination of membrane behaviors. Shape changes of liposomes have been observed when liposomes were subjected to different temperatures [4], osmolarities [5,6,7,8] and pHs [9,10], or when liposomes were treated with multivalent cations or detergents [5,6,11,12,13,14,15], and their detailed processes and mechanisms are still being intensively investigated. Liposomes have also been widely studied as a simple model system of biological membranes because the lipid bilayer is a basic component of biological membranes such as the cell membrane [16]. During the course of study, it has been revealed that cytoskeleton proteins, such as actin and tubulin, when encapsulated in liposomes, can generate forces sufficient to deform the lipid bilayer membrane by assembling into actin bundles or by polymerizing into microtubules (MTs) [17,18]. In addition, various peptides and proteins have been identified as membrane-deforming agents and deformation processes induced by them have been observed [19,20,21,22,23,24,25,26]. 

To better understand the morphological transformation of membrane vesicles, it is necessary to determine their mechanical properties, including bending stiffness and area compressibility. A number of experimental and theoretical studies have addressed these important issues using model vesicles such as artificial liposomes or living red blood cells [27,28,29,30,31,32]. Some studies have exploited optical tweezers as a tool to manipulate various sizes of vesicles ranging from nanometers to micrometers. 

**Figure 1:**
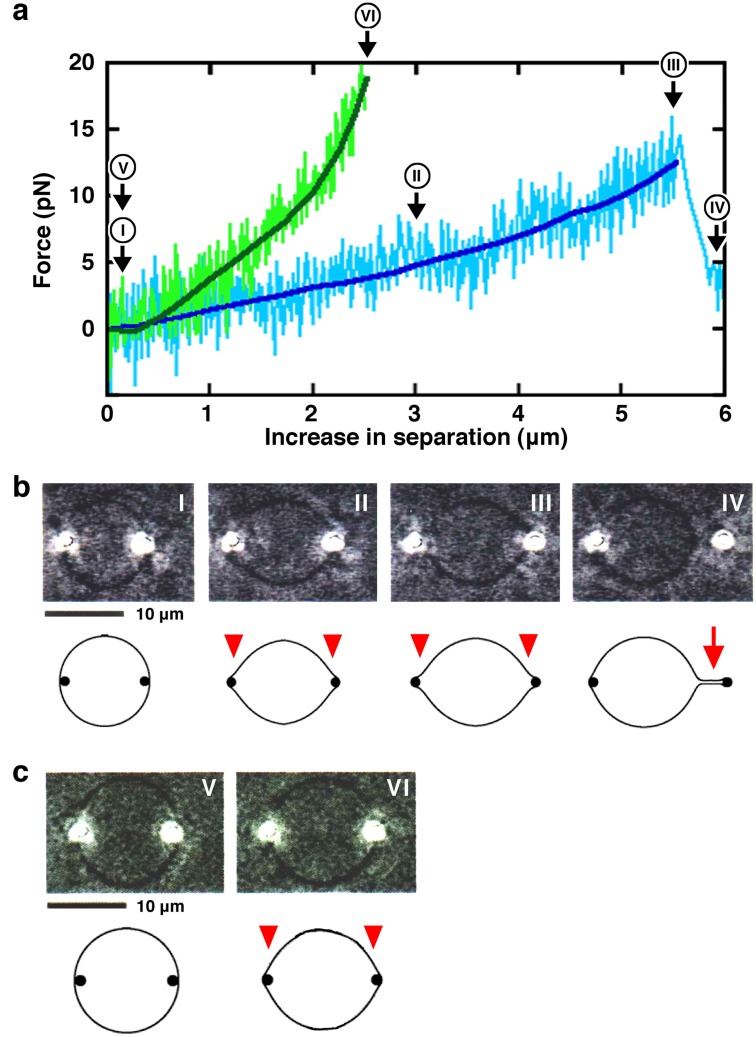
**(a)** Plot of the force exerted on a bead (vertical, pN) against the increase in separation between two beads encapsulated in a liposome (horizontal, μm). Two examples are shown, a case when the liposome forms a tubular membrane projection (blue) and a case when the bead moves out from the laser trap during liposome deformation (green). Each plot (thin line) was approximated by sixth-order equation (thick line); **(b)** and **(c)** Sequences of phase contrast images (each upper) and their models (each lower) showing the deformation processes of liposomes. Liposomes were made from phosphatidylcholine (PC) and phosphatidylglycerol (PG) obtained from native sources (4:1, mol/mol). Milli-Q water was used to swell the lipid films to prepare liposomes. Observations and measurements were carried out at 25 °C. In all experiments, to transform the liposomal membrane, separation between the two beads was increased at a constant speed of 125 nm/sec (refer to Section 3.3. for more information). (**b**) The process until just after the formation of a tubular projection from just after the initial elongation of a spherical liposome in a case when the liposome forms a projection. (**c**) The process until just before the moving out of the bead from the laser trap from just after the initial elongation of a spherical liposome. The images I–IV in (**b**) and V and VI in (**c**) are consistent with the symbols in (**a**). Bars indicate 10 μm. As one encapsulated polystyrene bead was moved away from the other one, a spherical giant unilamellar liposome (images I in (**b**) and V in (**c**)) formed two small bulge structures adjacent to both beads (images II and III in (**b**) and VI in (**c**)). Liposomes in this state are called lemon-shaped because of their morphology. In (**b**) and (**c**), the small bulge structures and the tubular membrane projection formed are indicated by arrowheads and an arrow, respectively.

In this study, to expand understanding of membrane behavior and to mimic a situation close to pseudopod formation in living cells, we measured the strength of force required to change liposome shape using double-beam laser tweezers [33]. Laser tweezers are a technique to manipulate fine particles using a laser beam [34,35]. The trapping force has a spring-like property, and the technique enables the measurement in piconewtons (pN) of force and nanometers (nm) of position accuracy, using the captured particles as probes. Two polystyrene beads (each 1 μm in diameter) were encapsulated in giant liposomes and were manipulated away from each other using the double laser beams. Without any specific interaction between the lipid membrane and the beads, mechanical forces can be applied to the liposome membrane from the inside. The mechanical force exerted by the two beads pushes the liposome membrane from within and transforms liposomes from spheres into lemon-like shapes that have two small bulge structures adjacent to the two beads. In the elongation stage of the transformation to lemon-shaped liposomes, the force required for the transformation became larger as the end-to-end length increased (Figure 1a). Subsequently, a tubular membrane projection was generated in the tip at either end (Figure 1b), or in some cases, the beads moved out from the laser trap without developing a tubular projection, probably because the repulsive force exerted on the beads exceeded the trapping force of the laser (Figure 1c). This process is similar to the liposomal transformation caused by the elongation of encapsulated cytoskeletons. The force reached a maximum strength (about 10–20 pN) just before a tubular membrane was generated. However, once the membrane tube developed, a decreased and constant force (about 4 pN) was required for further tube elongation or shortening. In the latter case, the force required for the membrane transformation reached a maximum strength (about 10–20 pN) just before the bead moved out from the laser trap. After the moving out of the bead, the liposomes returned to their spherical shape. These results indicate that the simple application of a mechanical force is sufficient to deform spherical liposomes and to form protrusions in the membrane. 

In this study, using the laser tweezers system, we examined the force required for the transformation of liposomes composed of different phospholipids or surrounded by different solutions. The results reveal that those parameters significantly affect the mechanical properties of membrane vesicles. Possible mechanisms by which the internal and external factors modulate the membrane transformability are discussed. 

## 2. Results and Discussion

We evaluated the mechanical properties of liposomes under various conditions using a double-beam laser tweezers system (Figure 1). When one polystyrene bead encapsulated in a spherical giant unilamellar liposome (Figure 1b I and 1c V) was moved away from the other “fixed” bead, the formation of small bulge structures adjacent to each bead was observed (Figure 1b II–III and 1c VI). A further force applied to the beads in these lemon-shaped liposomes subsequently generated tubular membrane projections (Figure 1b IV). The force exerted on the membrane was plotted against the change of its major axis length, *i.e.*, an increase in separation between the two encapsulated beads (Figure 1a, thin lines). The individual plots were approximated by sixth-order equation for the simple reasons of goodness of fitting (Figure 1a, thick lines). Hereinafter, the graphs in the Figures show plots that were approximated in this way. Processes from the initial spherical-shaped state until just before the formation of a tubular membrane projection (Figure 1b) or just before the moving out of the bead from the laser trap (Figure 1c) were plotted. Those data are expected to include the state in which the force required for deformation of the liposome has reached the maximum, and the results obtained under different conditions were compared to each other. The results obtained from liposomes with higher rigidity or elasticity tend to be plotted toward the upper left region of the graph, and inversely, those from liposomes with lower rigidity or elasticity tend to be plotted toward the lower right region. We designate liposomes in the former and latter cases as ‘hard’ and ‘soft’ liposomes, respectively. It should be noted that the solutions inside and outside of the liposomes prepared by the natural swelling of lipid films, the method employed in this study, are the same, and thus there is no difference of osmotic pressure. 

The results obtained as described above contain two cases; (1) liposomes formed tubular projections; or (2) the beads moved out from the laser trap during the liposome deformation (in this case, projections were not formed). Thus, in case (2) the maximum force shown in a plot (the end point of the plot in almost all cases) does not always indicate the strength needed to form a tubular projection. Data obtained from case (2) are also helpful to know the characteristics of the changes in force required for the deformation of liposomes and hence are informative to discuss the mechanical properties of liposomes. Therefore, based on the measurement results summarized as noted above, we will continue to discuss the effects of lipid composition and solution conditions on the mechanical properties of liposomes.

### 2.1. Effect of Lipid Composition 

The features of a lipid bilayer membrane depend on its lipid composition. In this study, we examined the effects of: (i) acidic phospholipid, by changing the mixing ratio between acidic phosphatidylglycerol (PG) and neutral phosphatidylcholine (PC); (ii) the size of the hydrophilic head group of lipid molecules, by substitution of the lipid used between PC and phosphatidylethanolamine (PE) or between PG and phosphatidic acid (PA); and (iii) the hydrophobic tail of lipid molecules, using a synthetic lipid possessing uniform fatty chain tails instead of natural lipids, on the mechanical properties of liposomes. 

**Figure 2:**
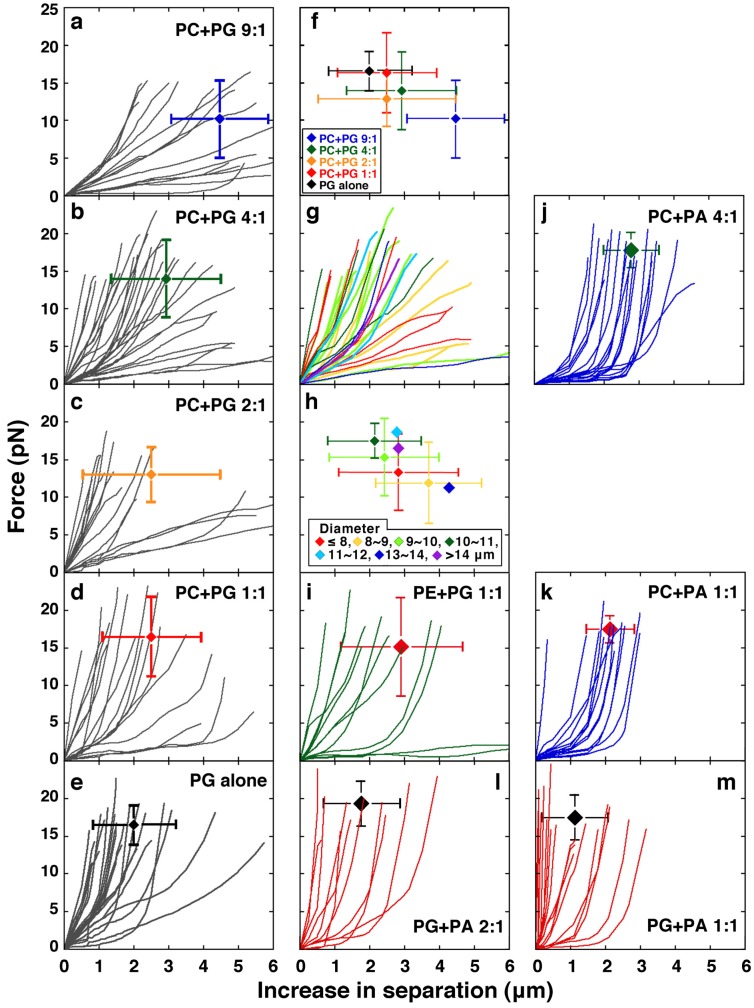
Effects of lipid composition on the mechanical properties of liposomes. (**a**)–(**d**) Results obtained from liposomes made from PC and PG; mixing ratios (mol/mol) of PC and PG are 9:1 (**a**); 4:1 (**b**); 2:1 (**c**) and 1:1 (**d**). (**e**) Results obtained from liposomes made from PG alone. From (a) to (e), the average and S.D. of the positions of end points of the plots in each panel is also indicated. (**f**) In order to investigate the dependency of the mechanical properties of liposomes against the content of PG, the average and S.D. shown in panels (**a**) to (**e**) are redrawn together with different colors (also see Supplemental Figure S1). The correspondence of color and composition of PC and PG is indicated at the bottom left of the panel. Between the case when the mixing ratio of PC and PG was 9:1 and other cases, in the increasing distance required for the projection formation or the moving out of the bead, p-values of t-tests are <0.005. (**g**) In order to investigate the effect of liposome size, the results shown in (**b**) are redrawn. Each plot is colored according to the diameter of the corresponding liposome. The correspondence of color and the range of liposome diameter is indicated at the bottom of panel (**h**). (**h**) For each range of liposome diameter, the average and S.D. of the positions of end points of the plots shown in (**g**) are obtained and indicated by the different colors. In the case when the number of the plots is <3, only the average is indicated (also see Supplemental Figure S2). (**i**) Results obtained from liposomes made from PE and PG (1:1, mol/mol). (**j**) and (**k**) Results obtained from liposomes made from PC and PA; the mixing ratios (mol/mol) of PC and PA are 4:1 (**j**) and 1:1 (**k**). (**l**) and (**m**) Results obtained from liposomes made from PG and PA. Mixing ratios (mol/mol) of PG and PA are 2:1 (**l**) and 1:1 (**m**). In (**i**)–(**m**), the average and S.D. of the ending positions of plots shown in each panel are indicated. In each condition, the number of the plots (N) is 16 (**a**); 30 (**b**); 16 (**c**); 17 (**d**); 22 (**e**); 12 (**i**); 19 (**j**); 13 (**k**); 11 (**l**); or 17 (**m**). Milli-Q water was used to swell the lipid films to prepare liposomes. All phospholipids used were obtained from native sources [13,24,25]. Measurements were carried out at 25 °C. The average and S.D. of the ending positions of plots shown in all panels are shown in Supplemental Table S1.

It should be noted that the softness or hardness of liposomes is independent of their size as described later (see Figure 2g and h, and Supplemental Figure S2). Concerning the lipid compositions used for measurement in this study, the size distribution of liposomes prepared is not affected much by the lipid composition (*e.g.*, mixing ratio of PC and PG). Moreover, for the measurements, we used liposomes of a specific size range (about 5–15 μm in diameter). 

#### 2.1.1. Effect of Acidic Phospholipids

The lipid composition of PC alone is not suitable for experiments using laser tweezers because the low efficiency of forming liposomes that have a size suitable for manipulation and observation, the low efficiency of bead encapsulation and the high irregularity of their shapes makes the determination and measurement of the axis difficult. Therefore, the results obtained from liposomes made from PC and PG (mixing ratios of 9:1 to 1:1, mol/mol) and PG alone were compared (Figure 2a–e) in order to investigate the effects of an acidic phospholipid on the mechanical properties of liposomes. We used PG as a representative acidic phospholipid because PG has been frequently used experimentally, while biologically PG is commonly found in membranes of bacteria but not in membranes of eukaryotic cells. 

Even when the content of PG is low, the PG-containing liposomes formed tend to be spherical, which is attributable to the repulsive force exerted in the membrane due to the negative charge of PG. It is possible that the repulsive force alters the mechanical features of the membrane. In order to investigate the dependency of the mechanical properties of liposomes on the content of PG, the average and S.D. of the maximum force and the maximum increased distance obtained from each composition of PC and PG (as shown in panels (a) to (e) of Figure 2) are indicated in Figure 2f and in Supplemental Figure S1. As expected, with an increasing content of PG, liposomes had a tendency to be difficult to deform, that is, the tension force exerted in the membrane reached a larger strength by a shorter length elongation of the liposome. 

It is noted here that the lipid composition of PC and PG (4:1, mol/mol) is the most favorable to efficiently obtain spherical giant unilamellar liposomes encapsulating two beads, which are suitable for experiments using laser tweezers [33]. In other combinations of lipids, the samples prepared were occasionally unsuitable for measurement even when the liposomes formed had a spherical shape and convenient size, because encapsulation of the beads did not occur or too many beads were encapsulated. The balance between rates of the process by which the lipid bilayer membrane is peeled off from the lipid film and closes to form a liposome, and the process of incorporation into liposomes of the particles present in the solution added, is important. In this study, the number of plots shown in Figure 2b, which shows liposomes made from PC and PG (4:1, mol/mol), is the largest. Therefore, to investigate the effect of the size of liposomes on their mechanical properties, the results of Figure 2b were modified according to the diameter of liposomes, as shown in Figure 2g and h and in Supplemental Figure S2. Those results reveal that size has no significant effect. For a given elongation, the change in aspect ratio for liposomes with smaller sizes is much higher than that for liposomes with larger sizes. Therefore, the overall increase in surface area and tension should vary significantly with liposome size if liposome volume is unchanged and one might expect that the liposome size would affect the results. Surprisingly, the results obtained did not support that. It has been revealed that a base region which has a certain length is required to form a stable protrusion [36]. When forming a tubular projection by pressing from within, local events that occur around the bead to form such a base region connecting between the spherical and tubular parts in the membrane, rather than the deformation of the whole liposome, may be important. 

We have previously found that liposomes containing an acidic phospholipid such as PG have a tendency to become spherical compared to liposomes composed only of neutral lipids [13,37]. PG molecules present in the liposome membrane may repel each other [38], *i.e.*, the repulsive force between PG molecules caused by their negative charges maximizes their distance from each other and stabilizes the morphology of closed vesicles to the most stable spherical shape. Moreover, the electric repulsive force may overcome the membrane fluctuation due to the Brownian motion of surrounding molecules, such as water. Thus, when PG is contained in the membrane, it can be assumed that larger forces are required to deform the liposomes. If the surface area is constant, the inner volume of a liposome decreases with the progress of the protruding transformation of spherical liposomes. In other words, the distance between different parts of the liposome decreases with elongation of the major axis. As a result, the repulsive force, which is important for the membrane tension, should be increased due not only to the bending elasticity but also to the electrostatic interaction. Consistently, many liposomes increase the force strength rapidly from the middle of the process. Most liposomes made from PC and PG are spherical. However, depending on the mixing ratio between PC and PG, the liposomes have different mechanical features (Figure 2a–f). These results suggest that, even if liposomes have the same spherical shape, they have different elasticities due to differences in the repulsive force acting in the membranes. Conversely, liposomes that do not possess a charge, such as those made from PC alone, are thought to be soft and to fluctuate by Brownian motion because the repulsive force between the membranes is weak. 

Interestingly, liposomes appear to be mixes of two groups, stiff and very easily elongated ones (see Figure 2c as a representative result). Heterogeneity in the degree of mixing of lipids between PC and PG that may take place during the swelling of a lipid film is a reason for the existence of two groups. Easily elongated liposomes, however, were also occasionally present among liposomes made from PG alone (Figure 2e). Since both the PC and PG used were obtained from native sources, they have heterogeneous fatty acyl chains as hydrophobic tails. This heterogeneity in the hydrophobic tails of the lipids is a possible reason. Moreover, identical results were not always obtained even when the experiment was repeated using the same liposome, suggesting that there is a non-uniformity in liposome membranes that may result from heterogeneities derived from differences in the alignment or packing of constituent lipid molecules. If there is such a non-uniformity in liposome membranes, the results obtained will vary depending on the state of the region of the membrane that the beads contact. As another reason, the lamellarity of the membrane can be considered as a cause of the variation in force strength required for liposome deformation. However, it has been revealed that the majority of liposomes made are unilamellar [37,39,40]. In addition, suspicious liposomes, which appear as double- or multi-lamellar in the microscopic images, are eliminated from the measurement [33]. Even if multilamellar liposomes are included with the measured ones, it should show the results of hard liposomes and is not a reason for the presence of very easily elongated liposomes. 

#### 2.1.2. Comparisons between PC and PE and Between PG and PA, the Effect of Substitution with a Small Head Phospholipid

PE is a phospholipid where choline at the hydrophilic head part of PC is replaced with ethanolamine. PA is a phospholipid where glycerol at the hydrophilic head part of PG is replaced with hydrogen. Both PA and PG are acidic phospholipids and are negatively charged similarly in neutral pH conditions. Compared with PC and PG, PE and PA respectively possess smaller head parts, and thus PE and PA are designated as representatives of cone-shaped phospholipids. Therefore, we studied the effects of the molecular shape of phospholipids on the mechanical properties of membranes using liposomes containing PE or PA. 

As shown in Figure 2i, the properties obtained for liposomes made from PE and PG (1:1, mol/mol), both the increasing distance and the force strength required for the projection formation or for the moving out of the bead in these liposomes, are indistinguishable from those of liposomes made from PC and PG (1:1, mol/mol). In addition, when an acidic phospholipid PA was incorporated in the membrane, unlike the case of PE, easily elongated liposomes were not observed (Figure 2d,i, and m). These results suggest that PE has a similar effect on the mechanical properties of membranes to the neutral phospholipid PC, although PE is thought to be a weakly acidic phospholipid. Thus, PE seems to behave like a neutral phospholipid rather than a weakly acidic phospholipid, at least under the experimental conditions used here. 

By substitution of PG with PA, a slight tendency to harden liposomes was observed, that is, a larger force will be required for a shorter elongation. Very easily elongated liposomes, which occasionally exist in liposomes prepared from PC and PG, could not be observed in liposomes prepared from PC and PA (Figure 2j and k). In the case of liposomes made from PC and PA (4:1, mol/mol), the mean of the force strength required for the projection formation or the moving out of the bead (*i.e.*, the maximum force strength) becomes slightly larger than for liposomes made from PC and PG (4:1, mol/mol) (Figure 2b and j, p < 0.005). In addition, the mean of the maximum force strength in liposomes made from PG and PA (2:1, mol/mol) is slightly larger than in liposomes made from PG alone (Figure 2e and l, p < 0.05). Easily elongated liposomes, which rarely exist in liposomes prepared from PG alone, could not be observed at all in liposomes containing PA (Figure 2l and m). However, a significant difference was not observed statistically overall, even when PG was replaced with PA (compare Figure 2b and j, Figure 2d and k, and Figure 2e and m). Taken together, the results demonstrate that PA obtained from native sources is likely to form homogeneous and slightly hard membranes compared with PG. 

Generally, the size of the hydrophilic head of a phospholipid determines its molecular shape, and is an important factor that affects the curvature or morphology of the lipid bilayer. Because PC has a large head, the area occupied by the head of PC in the membrane surface becomes large, and the hydrophobic effect of fatty chain tails of PC is thought to be weakened [41,42]. These considerations suggest that differences in the size of the hydrophilic head will have a significant impact on the mechanical properties of membranes. As another mechanism, the head size of a phospholipid influences its packing density in the membrane, possibly resulting in differences in the permeation of water molecules through the membrane. The rate of water leakage from liposomes that takes place during the shape changes should influence the increase of force strength required for the shape changes. The results obtained reveal that the size of the hydrophilic head of a phospholipid molecule affects not only the morphology but also the mechanical properties of the lipid membrane. 

#### 2.1.3. Liposomes Made from Dimyristoylphosphatidylcholine (DMPC)

**Figure 3:**
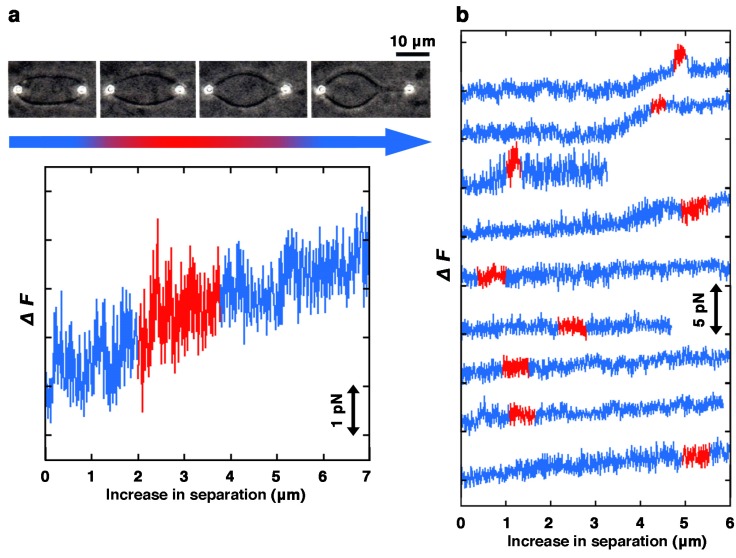
Measurements of liposomes prepared from DMPC. (**a**) Typical example of the process of membrane protrusion formation. Upper, sequence of phase contrast images showing a liposome being pulled. In this case, prolate or ellipsoidal liposomes were used for the measurement because spherical liposomes were difficult to prepare with DMPC. Bottom, change in the force strength required for the deformation. The color of the band below the photographs and the color of the line in the graph indicate the correspondence between images and graphs. Bar indicates 10 μm; (**b**) Results from nine different DMPC liposomes are shown in a single graph. From the time when the long axis starts to change (drawn in blue), through when the liposome is deformed to form a projection (red), until the projection is further elongated (blue), changes in the force strength required to deform the liposome are shown. Milli-Q water was used for swelling the lipid films to prepare liposomes. Observation and measurement were carried out at 25 °C.

Giant liposomes containing beads were obtained by the natural swelling of lipid films because of both the simplicity of liposome preparation and the ease of handling when the samples were manipulated with the laser tweezers. When the lipid composition was a single synthetic phospholipid that possessed uniform fatty chains, such as dilauroylphosphatidylcholine (DLPC), dimyristoylphosphatidylglycerol (DMPG) or dipalmitoylphosphatidylglycerol (DPPG), liposomes were not formed (data not shown). When the lipid composition was dipalmitoylphosphatidylcholine (DPPC) alone or distearoylphosphatidylcholine (DSPC) alone, only insoluble structures thought to be lipid aggregations were formed. The synthetic phospholipids that possessed uniform fatty chains that were available for liposome preparation included dimyristoylphosphatidylcholine (DMPC), dioleoylphosphatidylcholine (DOPC) and distearoylphosphatidylglycerol (DSPG) (Supplemental Figure S3). Almost all liposomes made from DMPC fluctuated without a predetermined shape. DOPC formed lipid droplets and liposomes. Liposomes made from DOPC had a variety of shapes, e.g., pearl necklace-, string- or peanut-like shapes similar to those made from natural PC. DSPG formed lipid aggregates and very small spherical liposomes. Therefore, with a single synthetic phospholipid possessing uniform fatty chains, liposomes that encapsulated beads were very hard to prepare. Only DMPC was able to form bead-encapsulating liposomes that could be manipulated by laser tweezers, even though they were not spherical. Unavoidably, we selected and used prolate or ellipsoidal liposomes for the force measurement (Figure 3). 

In the case of liposomes made from DMPC, all liposomes formed a membrane projection with an elongation force that was <5 pN without any resistive response (Figure 3). This strength of force is as small as half, or even smaller, compared to liposomes containing an acidic phospholipid that formed a projection (Figure 2, in almost all cases, >10 pN). This result indicates that DMPC liposomes are very easily elongated as expected from their morphology and behavior. There are two possibilities that might explain the observed ease of deformation of DMPC liposomes, the uniformity of the fatty chain tails or the absence of acidic phospholipids. Currently, we cannot determine which condition is more important for liposomes to be easily elongated. This is due to the fact that the results obtained from liposomes made from PC and PG (9:1, mol/mol) indicate that liposomes become soft with decreases in the content ratio of PG, whereas data concerning liposomes made from PC alone could not be obtained. The difference between liposomes containing an acidic phospholipid and those made from DMPC suggests that the mechanisms of tubular projection formation are different from each other. The structure of the lipid bilayer, packing and/or alignment of lipid molecules at the transition region between the tubular projection and the spherical part of the liposomes might be different. 

In this study, liposomes composed of a single phospholipid that were available for measurement, were only made from DMPC. Therefore, the effect of hydrophobic tails of phospholipids, their chain length and degree of unsaturation, on the mechanical properties of liposomes could not be compared. However, the morphology and behavior, such as membrane fluctuation, of liposomes observed by dark-field microscopy strongly suggest that liposomes possess various mechanical properties depending on their lipid composition (Figure 2 and Figure 3, and Supplemental Figure S3). Properties, such as phase transition temperature, of many membranes composed of a single PC species have been reported [41,42]. The phase transition temperature of membranes made from DMPC is about 24 °C, and is close to the temperature at which the measurement is performed [43]. Since it has been reported that the bending rigidity of membranes significantly drops while the permeability to water increases at the transition temperature [44], this is a considerable reason why liposomes made from DMPC are very easily elongated. 

In addition, using prolate or ellipsoidal liposomes for the measurement is also a considerable reason for the lower stiffness of liposomes made from DMPC. If the shapes of liposomes are different, liposomes will have different stiffness. When being stretched from two poles, the prolate or ellipsoidal liposomes are expected to possess lower stiffness than the spherical liposomes since non-spherical liposomes, but not spherical ones, can change shape without increasing surface area [30,31]. 

If various liposomes that consist of a single phospholipid, *e.g.*, liposomes made from DOPC alone or DSPG alone, were available for measurement, the relationships between the properties and the membrane composition could be further elucidated. 

### 2.2. Effect of Solution Conditions

In order to control the pH of the solution and to prevent denaturation of the protein(s) added, the following experiments were carried out in solution with a buffer. It should be noted that in every experiment in this study, the solutions inside and outside of the liposomes are the same. Therefore, we assume that there is no membrane potential in the liposome membranes used.

#### 2.2.1. Effect of pH

**Figure 4:**
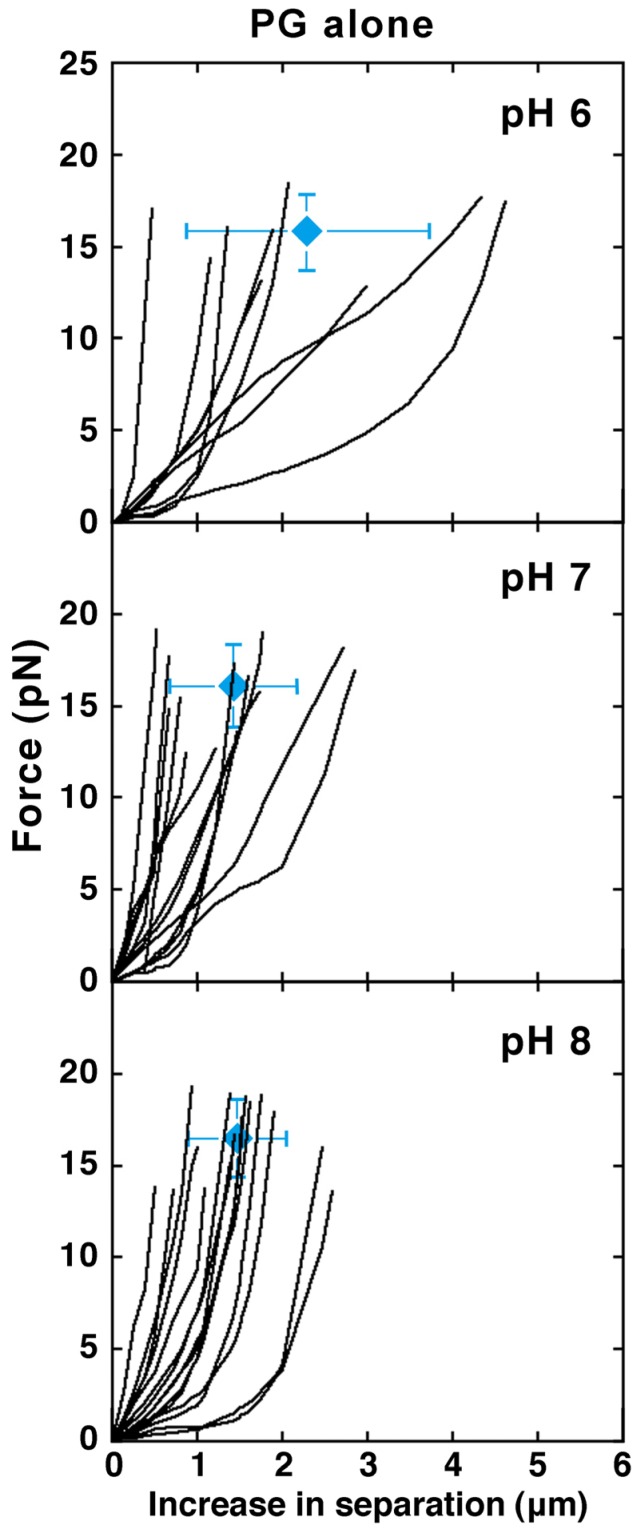
Liposomes made from PG alone under different pH conditions. Top, middle and bottom show the results for pH 6, 7 and 8, respectively. In each panel, the average and S.D. of the ending positions of plots is indicated (N is 9 (pH 6), 13 (pH 7) and 15 (pH 8)). The numerical of the average and S.D. is shown in Supplemental Table S2. HEPES-buffer (5 mM HEPES-NaOH, pH 7.0), HEPES (pH 8.0)-buffer (5 mM HEPES-NaOH, pH 8.0) or MES-buffer (5 mM MES-NaOH, pH 6.0) were used for swelling the lipid films to prepare liposomes. The PG used was obtained from native sources. Measurements were carried out at 25 °C.

To investigate the effects of pH on the mechanical properties of lipid bilayer membranes, we manipulated liposomes made from PG alone, which is the lipid composition that seems to be susceptible to pH, at pH 6, 7 and 8 (Figure 4). In the pH range 6–8, most liposomes prepared were spherical and available for the experimentation. The results of the mechanical measurements showed that softer liposomes were produced at pH 6 compared with pH 7 and 8. In most liposomes at pH 6, when the major axis was extended several μm, the force required for the deformation finally reached 15 pN. A significant difference was not observed between pH 7 and 8, and in both cases, the force required for the deformation exceeded 15 pN when the major axis was extended around 1 μm. In the above experiments, however, no significant differences were found between the condition of pH 6 and the condition of pH 7 or 8. 

When the pH was higher than the acid dissociation constant of PG, the phosphate group of PG is deprotonated and PG has a negative charge. At pH 6, PG is somewhat neutralized. By losing the negative charge, the surface charge of PG-containing liposomes also decreases. Consequently, the repulsive force in the membrane is weakened compared with the cases of pH 7 and 8, and the liposomes become soft. There is no difference in the results between pH 7 and 8, which is attributable to the fact that PG has a negative charge identically at those pH conditions. On the other hand, it has been known that the membranes of liposomes possessing negative charges, such as PG-containing liposomes, have changes in their fluidity, the hydration state of the surface or the lipid interactions within them depending on the pH of the solution. It has been known that interactions between the hydrophilic heads of the composing phospholipids are increased by hydrogen bond formation when the pH is low [45]. If this effect is larger than that of the canceling of negative charges that takes place at lower pH, the membrane will become hard. At least in the pH range 6–8, the effect of the pH of the environment solution on the mechanical properties of liposomes may be much smaller than those of changing the net charges of the phospholipid or the shapes of the phospholipid molecules, *i.e.*, the size of the hydrophilic head or the length and degree of unsaturation of the hydrophobic tails. In this study, for reasons of the efficiency of liposome preparation, we did not obtain results at much lower pH conditions. If experiments could be carried out at a pH lower than 6.0, there is a possibility that more significant differences, for example, the softening of liposomes due to cancellation of the negative charge of the acidic PG, would be observed. In order to investigate the effect of pH of the solution, it is necessary to develop experimental conditions that would allow for more varied lipid compositions and a wider range of pH to compare results.

#### 2.2.2. Effect of Proteins in the Solution

A wide variety of proteins functions in living cells. It is well known that transmembrane or membrane-bound proteins affect the properties of lipid membranes. On the other hand, it has not been well studied yet how the behavior of the lipid bilayer membrane is changed by a soluble protein contained in the solution surrounding the membrane. Here, we examined the mechanical properties of liposomes in the presence of proteins that do not bind to the membrane. It should be noted that, since the buffer containing the protein was added to the lipid film in order to prepare liposomes and thus the inside and outside solutions were the same, osmotic stress need not be taken into consideration. 

**Figure 5:**
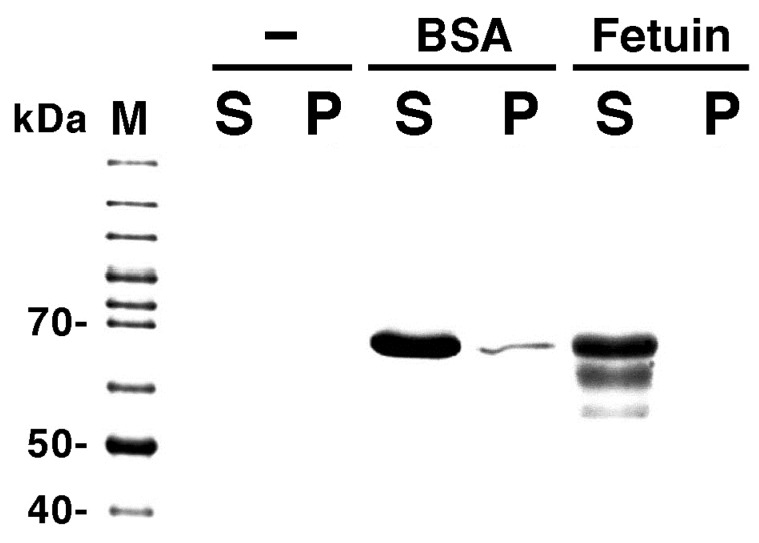
Cosedimentation assays of proteins with liposomes. ‘−’, ‘BSA’, and ‘Fetuin’ indicate the protein assayed (‘−’ indicates that a solution containing liposomes made from PC and PG (1:1, mol/mol) was centrifuged in the absence of protein). ‘M’, ‘S’ and ‘P’ indicate molecular weight markers, the supernatants and pellets of the centrifuged samples, respectively. All phospholipids used were obtained from native sources. HEPES-buffer alone or buffer containing 2.0 mg/mL BSA or 0.2 mg/mL fetuin was used for swelling the lipid films to prepare liposomes. The molecular weight of BSA is 66 kDa. It should be noted here that, even though the molecular weight of fetuin is about 50 kDa, this protein usually appears on the gel at the position of 50–70 kDa [47]. In the experimental conditions, if proteins bound to liposome membranes only weakly with a dissociation constant of the micro molar order, yet almost all proteins should be co-sedimentated to the precipitate fraction. However, only a very slight band of BSA and no band of Fetuin were detected in the precipitate fractions. Thus, we conclude that these proteins have little or no binding affinity to the membrane.

As proteins that do not bind to the lipid membrane, bovine serum albumin (BSA) and fetuin were used. We confirmed that BSA and fetuin do not bind to liposomes using a cosedimentation assay (Figure 5). BSA does not bind to liposomes, regardless of their lipid composition. Therefore, BSA is a protein that has been used as a control for cosedimentation assays between proteins and liposomes frequently used in our studies [17,21]. Fetuin, which is also obtained from bovine serum, is an acidic protein that is involved in the regulation of mineralization and bone formation [46]. Therefore, the electrostatic repulsion is one major reason why fetuin does not bind to liposomes containing acidic phospholipids, such as liposomes made from PC and PG. 

**Figure 6:**
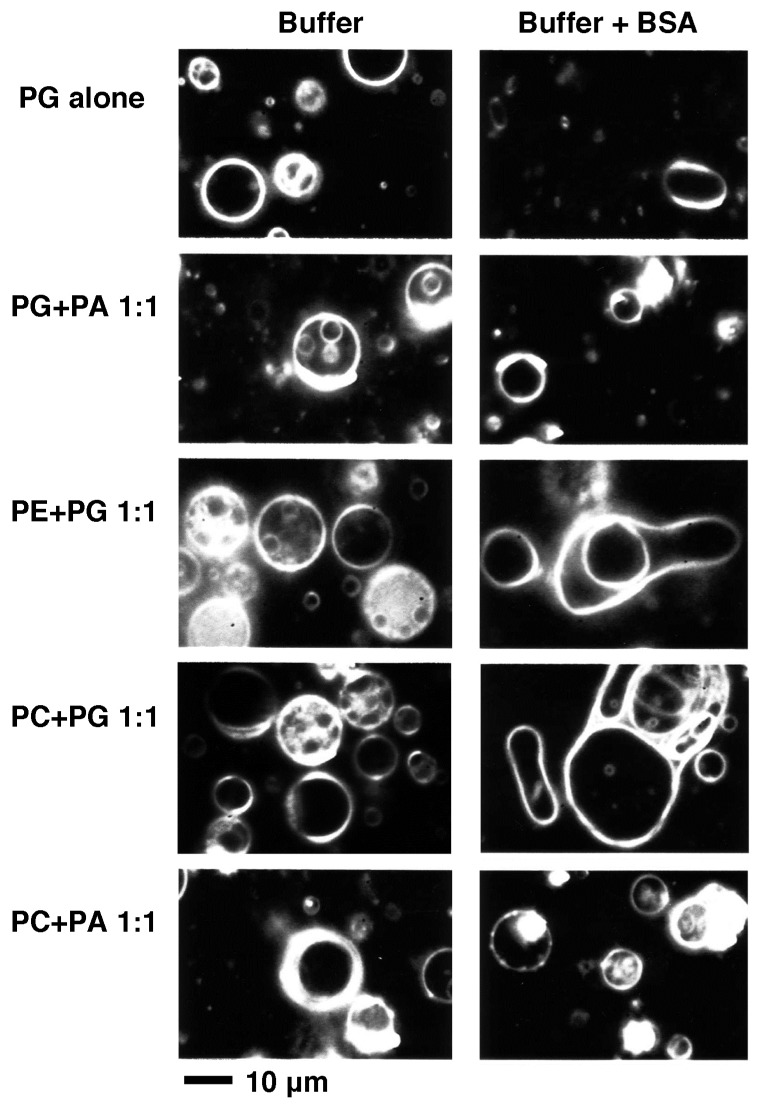
Dark-field images of liposomes prepared in the presence (right column) or absence (left column) of BSA. The lipid composition is indicated on the left of each row. HEPES-buffer with or without 2.0 mg/mL (30 μM) BSA was used for swelling the lipid films to prepare liposomes. All phospholipids used here were obtained from native sources. Bar indicates 10 μm. Observations were carried out at 25 °C.

**Figure 7:**
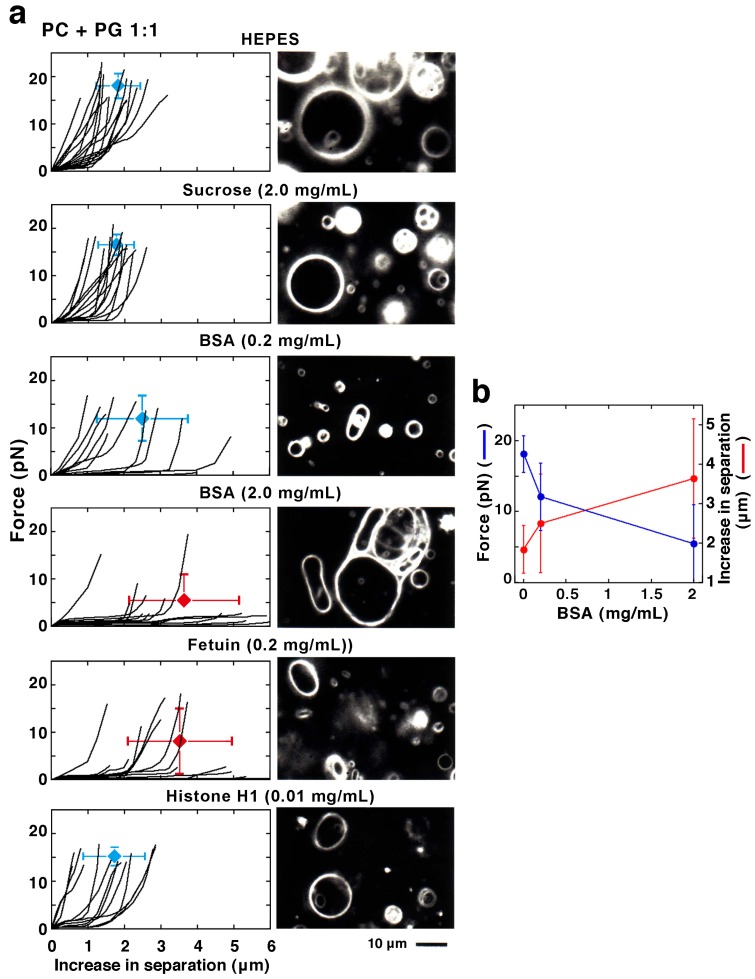
(**a**) Plotted results (left) and dark-field images (right) of liposomes prepared from PC and PG (1:1, mol/mol). The condition of solution, the solute and its concentration is indicated at the top of each row. HEPES-buffer with or without BSA, fetuin, Histone H1 or sucrose was used for swelling the lipid films to prepare liposomes. The PC and PG used were obtained from native sources. In each plot, the average and S.D. of the ending positions of plots are indicated (N is 14 (HEPES-buffer alone, control), 15 (2.0 mg/mL (5.8 mM) sucrose), 11 (0.2 mg/mL (3 μM) BSA), 14 (2.0 mg/mL (30 μM) BSA), 13 (0.2 mg/mL (4 μM) fetuin) and 13 (0.01 mg/mL (0.5 μM) Histone H1)). The numerical of the average and S.D. is shown in Supplemental Table S3. Between the control and the cases of liposomes with 2.0 mg/mL BSA or 0.2 mg/mL fetuin, p-values are <0.001 for the maximum force and <0.005 for the increasing distance required for formation of the projection or the moving out of the bead. Bar indicates 10 μm. Observations and measurements were carried out at 25 °C. Note that the dark-field image of liposomes with 2.0 mg/mL BSA is the same photograph used in Figure 6. (**b**) Average values of the maximum force and increase in separation are summarized against the BSA concentration. The data shown in graphs of the first, third and fourth panels of (**a**) are used.

Liposomes made from PG alone, PG and PA, PE and PG, PC and PG, and PC and PA (1:1 mol/mol) prepared using HEPES-buffer were spherical and stable (Figure 6, left column). When liposomes with the same lipid compositions were prepared using HEPES-buffer with 2.0 mg/mL BSA, in cases where the lipid composition contained PA, liposomes kept their stable spherical shape, but in the other cases, liposomes tended to show elongated unstable shapes and the majority of them were fluctuating their shapes (Figure 6, right column). Following the addition of BSA to the solution, liposomes were softened (Figure 7). That observation suggests that if the surrounding solution contains a protein, the behavior of liposomes will be affected even though the protein does not bind directly to the membrane. The difference in liposomal behavior between the cases of lipid compositions with or without PA may be attributable to the effect of PA that slightly hardens the membranes as described above (see Section 2.1.2, Figure 2e, and j–m). 

The effect of BSA that softens the liposomes is dependent on the protein concentration. Indeed, easily elongated liposomes increase in number in liposomes made from PC and PG (1:1 mol/mol) with increasing BSA concentration in the solution (Figure 7b, 0.2 and 2.0 mg/mL BSA). In particular, liposomes elongated along their major axis without any increased response (e.g., increase of the repulsive force), even when they were elongated a long distance. These results suggest that if the surrounding solution contains a protein, the mechanical properties of the membranes will be affected even though the protein does not bind directly to the membrane. The same softening effect could be observed with fetuin (Figure 7a, 0.2 mg/mL fetuin). Fetuin is a soluble serum protein possessing a similar molecular weight to BSA. For softening liposomes, 0.2 mg/mL fetuin has the same potency as 2.0 mg/mL BSA. 

On the other hand, even when much higher concentrations of sugar were added to the buffer used to prepare liposomes instead of a protein, no difference was found in either the mechanical properties or the shape changes of liposomes compared with cases when the buffer did not contain sugar (Figure 7a, 2.0 mg/mL sucrose). This result indicates that the softening effect is particular to the soluble proteins BSA and fetuin, and shows that the basis of this effect is not derived from a non-specific and common effect from the addition of a solute, as for example from the osmotic pressure. 

Proteins have polar and/or charged residues at their surface, and in some cases, they also have hydrophobic regions. Therefore, it is possible that proteins would exert some effect on the mechanical properties of lipid membranes via various types of interactions with the membrane surface, even in cases without specific binding. In other words, it is possible that liposomes become soft by cancellation of the effect of membrane charge that results from the disruption of the repulsive force or the shielding of the charge due to the charged proteins both inside and outside of the liposomes. For such cases, the association and dissociation to liposomes of proteins may be very rapid and would not be detected by a cosedimentation assay. There may be a weak binding interaction, which is not sufficient to be detected by the assay, between liposomes and BSA or fetuin. Therefore, a method that is highly sensitive and sufficient to detect binding between proteins and liposomes, for example FRET or surface plasmon analysis, will be required for further study.

#### 2.2.3. Effect of Membrane-Interacting Proteins in the Solution

Histone H1, a well-characterized DNA-packaging protein, is known to bind to membranes containing PG and to reduce the lateral diffusion of PG molecules in the membrane [48]. Therefore, as an example of a membrane-binding protein, we investigated the effects of histone H1 on the mechanical properties of liposomes containing PG. As described above, we prepared liposomes made from PC and PG (1:1 mol/mol) using a HEPES-buffer containing histone H1. However, lipids aggregated at concentrations at about 10 μM histone H1; thus, liposomes with high concentrations of histone H1 could not be obtained. Therefore, we prepared liposomes using lower concentrations—0.5 and 1 μM—of histone H1. Consequently, liposomes encapsulating beads suitable for study were obtained, but their number was few and we were unable to obtain a sufficient amount of data. The force required for the projection formation of spherical liposomes under conditions of 0.5 μM histone H1 was measured, and there was no difference compared with the case without histone H1 (Figure 7a, 0.01 mg/mL Histone H1). Thus, at the low concentrations used here, histone H1 does not seem to influence the membrane properties. Some liposomes were fluctuating their membranes when the concentration of histone H1 was 0.5 or 1 μM, which may be a consequence of the effect of the protein present in the surroundings as discussed above. 

### 2.3. Physiological Significance Related to Cytoskeletal Components

As described above, we found that the force required to deform cell-sized lipid vesicle membranes will take different strengths in the range from a few pN up to 30 pN depending on the lipid composition and solution condition. Actin filaments and MTs are cytoskeletons that actively maintain the morphology and movement of living cells. It has been reported that actin filaments and MTs are able to generate mechanical forces from a few pN to tens of pN, during their polymerization reaction [49,50,51,52,53,54]. The molecular motors, myosin, kinesin and dynein, are also known to generate forces of several pN by sliding along actin filaments or MTs [55,56]. Although the strengths are different between the forces able to be generated by the cytoskeletons and motors and those required for the deformation of membranes, they could be comparable. Taken together, our results suggest that the morphology of biological membranes could be regulated not only by the well studied systems of cytoskeletons and molecular motors but also by regulating the mechanical properties of lipid bilayer membranes by changing their constituent lipids and/or the environment of the neighboring solutions. According to this line of thought, prospectively, the effect of cholesterols or membrane proteins involved in linking between biological membranes and cytoskeletons on membrane properties should be further investigated using the experimental system discussed in the next section. 

### 2.4. Possible Collaboration with Other Methods and Overcoming the Limitations of This Study

In this study, there are restrictions on the conditions of the solutions and the lipid compositions of the membranes used for the following reasons: the liposome preparation by natural swelling, the necessity of encapsulating appropriate numbers of beads into liposomes, and the manipulation of the encapsulated beads by laser tweezers. For assumption of physiological conditions, salts, especially potassium and magnesium, are indispensable solutes. The compositions of membranes, not only phospholipids but also other constituents, such as glycolipids, cholesterols and integral membrane proteins, are involved in the regulation of fluidity and/or curvature of membranes through control of phase transition and domain formation in the membrane or leading asymmetry between two leaflets [57,58]. The reinforcement of biological membranes is thought to be the result of an interplay between the lipid bilayer and the underlying network of proteins such as those represented by cortical actin filaments and membrane-associating spectrin [59]. To further evaluate the effects of those factors, a measurement method with much less constraint is required to investigate the mechanical properties of lipid membranes. Methods for liposome preparation have continued to improve [60,61]. 

Note that the term “membrane mechanical properties” as used in this report reflects the combined effects of the different membrane elastic constants such as bending elasticity and membrane tension. In addition, the effects of water permeation or the detailed shape of the liposome on liposome deformation could not be excluded completely. Previously, the bending elasticity and tension of membranes have been successfully characterized separately using model vesicles [27,32], and the mechanical constants of red blood cells have been successfully revealed using the similar double-trap laser tweezers [28,29]. Also, studies based on theoretical computation have been energetically pursued [30,31]. 

By combining those improved and useful methods, direct measurements of the force acting on the liposome membrane could be made under a much wider range of conditions (*e.g.*, with cytoskeletal networks underlying the membrane and with a high concentration of molecules encapsulated that are similar to the cytosol). Such analyses would be helpful to understand the properties and behaviors of membranes during deformations such as tubulation. 

## 3. Experimental Section

### 3.1. Lipids and Other Materials

All phospholipids used were purchased from Sigma (St. Louis, MO, USA). BSA and fetuin isolated from bovine serum, Histone H1 and sucrose were purchased from Wako Pure Chemicals (Osaka, Japan). Beads (polystyrene beads, 0.992 μm diameter) were purchased from Polysciences (Warrington, PA, USA).

### 3.2. Preparation of Liposomes

Liposomes were prepared as described previously by the natural swelling of lipid films [13,21]. Phospholipids (10 mM) in a chloroform/methanol solution (98:2 (vol/vol)) were mixed in test tubes. The organic solvent was then evaporated under a flow of nitrogen gas, and the lipids were further dried in vacuo for at least 2 hours, and placed more than 12 hours in a desiccator in a vacuum. To form liposomes, a solution of Milli-Q water (pure water), HEPES-buffer (5 mM HEPES-NaOH, pH 7.0), HEPES (pH 8.0)-buffer (5 mM HEPES-NaOH, pH 8.0) or MES-buffer (5 mM MES-NaOH, pH 6.0), with or without the indicated proteins or sucrose, was added to the dried lipid films in the test tubes adjusting the concentration of total lipids to 1 mM in all cases, and then was incubated for 1 hour. During the incubation, the lipid films were spontaneously peeled off into the solution and subsequently closed to form liposomes. In principle, the solutions inside and outside the liposomes prepared by this method are the same, and thus there is no difference of osmotic pressure. It should be noted here that there is no significant difference in the experimental results between cases of Milli-Q water and the buffer used when liposomes were made from PG alone (Figure 2e and Figure 4, and Supplemental Tables S1 and S2), and among cases of Milli-Q water, the buffer, and the buffer containing sucrose used when liposomes were made from PC and PG (1:1, mol/mol) (Figure 2d and Figure 7 (control and 2 mg/mL sucrose), and Supplemental Tables S1 and S3). 

To form liposomes encapsulating beads, a solution containing 0.1–0.5% (weight/vol) of polystyrene beads was added to the dried lipid films as described above [33]. Beads not encapsulated in liposomes were removed as a precipitate by centrifugation (2000 g × 5 min). By this degree of centrifugation, the bead-encapsulating liposomes remained in the supernatant. 

Prior to manipulation of liposomes and measurement of forces required for the transformation by laser tweezers, the liposomes prepared were observed with a dark-field microscope (Hiapo, Olympus, Tokyo, Japan) incorporating a 200 W high-intensity mercury lamp (Osram, Munich, Germany), a 100× objective lens (PC, Olympus) and a condenser (BHF, Olympus). Preparation and observation of liposomes were carried out at room temperature. 

It should be noted that, unless the majority of liposomes prepared as described above were spherical, the liposome sample obtained was not used for measurement, except for liposomes prepared with DMPC, because those liposomes were usually non-spherical and highly irregular. 

### 3.3. Laser Tweezers

To measure liposomes using laser tweezers, we selected only spherical-shaped liposomes, and eliminated liposomes that had other shapes from the measurement as much as possible (except in the case of liposome prepared with DMPC as described above). Manipulation of beads in liposomes using the laser tweezers was carried out at 25 °C. The liposomes and beads were visualized in a phase-contrast microscope with built-in laser tweezers [33]. 

Laser tweezers were used as previously described [33,55]. An objective lens (ph-3 Plan Apochromat, 100×, N.A. = 1.4, Zeiss, Jena, Germany) was used to manipulate the beads with the beams from a YAG-laser (T10-V-106C, spectra-physics, Tokyo, Japan). Liposomes of about 5–15 μm in diameter that each contained just two beads were selected and were transformed by tension applied through the inner beads using the double-beam laser tweezers. Each bead was trapped in the focus of each beam, and the trapped beads were moved using galvano scanners. To transform the liposomal membrane, one beam was fixed in position, and the other one was moved at a constant speed of 125 nm/sec. Since the moving speed of the beads is faster than the discharge rate of the water, a rapid increase of forces has been observed. On the other hand, permeation to some extent of water also should have occurred, because the liposomal deformation itself should hardly occur if the water permeation does not occur. Previously, we repeated the increase and decrease of separation length between two beads to repeatedly deform the same liposome between a tubule projected shape and a sphere [33]. As a result, in the second or subsequent deformation cycles, we found both cases of: (i) the plot does not show any change from that obtained by the first deformation cycle; and (ii) the maximum force observed just before the tubule formation decreases. During the shape changes, the former is considered as the case when only a negligible amount of water permeated, and the latter is considered as the case when the liposome discharged water enough for slackening. The phase-contrast images of liposomal transformation and bead movement were visualized with an SIT camera (C2400, Hamamatsu Photonics, Hamamatsu, Japan). These images were enhanced by an image processor (ARGUS-10, Hamamatsu Photonics) and were recorded on a video recorder. 

The force strength applied to liposomes (about 5–15 μm in diameter) was estimated from the displacement distance of beads trapped in the fixed beam as described previously [33,55]. The images were transferred to a personal computer as sequential images (30 images/sec) and were analyzed. The bead position was determined as the center of brightness. The bead displacement from the laser center was determined and was proportional to the force applied to the bead. The trapping force was calibrated by measuring the Brownian motion of the bead. 

A bead trapped in the bulk solution indicates a displacement of about 10 nm in noise. Thereby, the accuracy of the force measurement by the laser trap is estimated as about 1 pN. Image contrasts have been changed according to the purpose, and thus, the contrast is different between the cases for analysis and for presenting pictures. In the former case, beads were seen as gray, whereas, in the latter case, there is a halo around the bead because the contrast has been emphasized in order to make the liposome membranes visible. 

### 3.4. Cosedimentation Assay

The cosedimentation of proteins with liposomes was performed as described previously [21] with slight modification. A mixture of HEPES-buffer containing liposomes prepared as described in Section 3.2 (final 1 mM lipids, PC and PG (1:1, mol/mol)) and the same buffer containing final 2 mg/mL BSA or final 0.2 mg/mL fetuin were centrifuged at 80,000 ×g for 1 hour at 25 °C. The separated samples were resolved using SDS-PAGE by applying the samples to 12.5% gels. Protein bands were stained with CBB. 

## 4. Conclusions 

This study succeeded in quantifying the mechanical properties of cell-sized giant unilamellar liposomes by manipulating beads encapsulated within them using laser tweezers. The results revealed that the properties of the liposomes were drastically affected by the lipid composition of the membrane and the solution environment. As expected, the mechanical properties depend on the charge and shape of the composing phospholipid molecules. Surprisingly, the mechanical properties of liposomes could be changed even when a protein, which shows no or little interaction with the membrane, is present in the solution. The force required to deform liposomes takes a different strength in the range from a few pN up to 30 pN depending on the lipid composition and solution conditions, which are comparable to forces that can be generated by polymerizing cytoskeletons and molecular motors in living cells. The results obtained suggest that the mechanical properties of cells or organelles that are made with the biological membranes could be much more dynamically regulated by a number of factors provided *in vivo*. In elucidating the dynamic mechanism(s) determining the morphogenesis of lipid membrane vesicles both *in vitro* and *in vivo*, these findings provide important clues. Directly measuring the force acting on the liposome membrane as reported here is a powerful analytical method to unveil the physicochemical properties of lipid membrane vesicles, and furthermore of cells and organelles. 

## Supplementary Files

Supplementary File 1
