# Supplementary material for: Effects of Lipid Composition and Solution Conditions on the Mechanical Properties of Membrane Vesicles
Source: Membranes (Basel). 2015 Jan 20;5(1):22–47. doi: 10.3390/membranes5010022 (PMC4384090; doi:10.3390/membranes5010022)
Supplement: Supplementary File 1 [file membranes-05-00022-s001.pdf]

Supplementary Materials

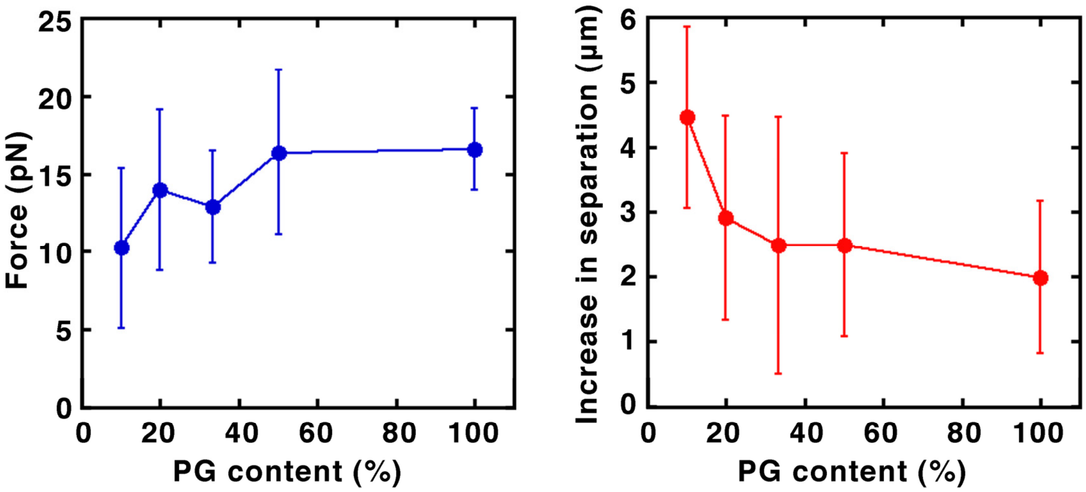

Figure S1. The average values of the maximum force (left) and the increase in separation (right) are plotted against the content of PG. Error bars indicate S.D. Results shown are from Figure 2f.

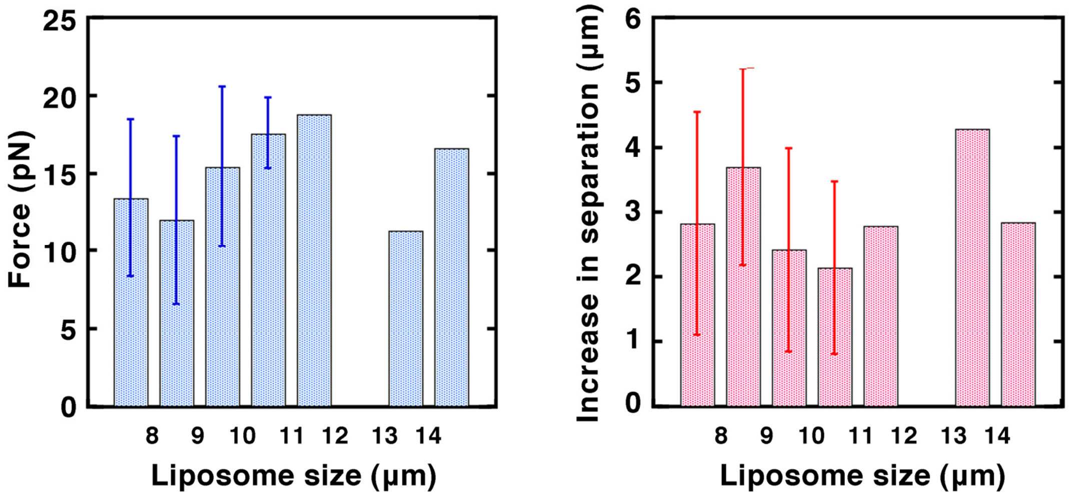

Figure S2. The average values of the maximum force (left) and the increase in separation (right) are plotted against each range of liposome size. Error bars indicate S.D. In cases when the number of results is <3, only the average is indicated. Results shown are from Figure 2h.

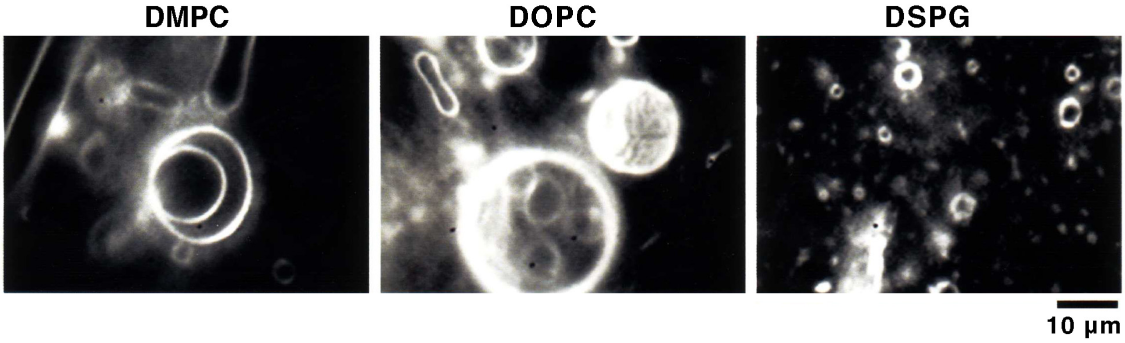

Figure S3. Dark field images of liposomes, or droplets or aggregates of lipids, that were formed from single synthetic phospholipids (in order of DMPC, DOPC and DSPG as noted). Milli-Q water was used for swelling the lipid films to prepare liposomes. Bar indicates 10 μm. Observations were carried out at 25 °C.

Table S1. The average and S.D. of the maximum force and the expanding distance required for the projection formation or the bead deviation. The effect of lipid composition is shown. Milli-Q water was used.

Phospholipid	Mixing ratio (%)	The maximum force strength (pN)	Increase in separation (μm)
PC + PG	9:1	10.3 ± 5.1	4.5 ± 1.4 (c)
	4:1	14.0 ± 5.2 (a)	2.9 ± 1.6 (c)
	2:1	12.9 ± 3.6	2.5 ± 2.0
	1:1	16.4 ± 5.3	2.5 ± 1.4
PG alone		16.6 ± 2.6 (b)	2.0 ± 1.2
PE + PG	1:1	15.2 ± 6.5	2.9 ± 1.8
PC + PA	4:1	17.7 ± 2.3 (a)	2.8 ± 0.8
	1:1	17.5 ± 1.7	2.1 ± 0.7
PG + PA	2:1	19.4 ± 3.0 (b)	1.8 ± 1.1
	1:1	17.5 ± 3.0	1.1 ± 1.0

Notes: *p*-values are <0.005 (a), <0.05 (b) and <0.005 (c).

Table S2. The average and S.D. of the maximum force and the expanding distance. The effect of pH is shown. Liposomes examined here were made from PG.

pH	The maximum force strength (pN)	Increase in separation (μm)
6	15.9 ± 2.0	2.3 ± 1.4
7	16.1 ± 2.2	1.4 ± 0.8
8	16.6 ± 2.2	1.5 ± 0.6

Table S3. The average and S.D. of the maximum force and the expanding distance. The effect of the protein solubled in the solution is shown. Liposomes examined here were made from PC and PG (1:1, mol/mol). HEPES-buffer was used.

Solute	Concentration (mg/mL)	The maximum force strength (pN)	Increase in separation (μm)
Control		18.1 ± 2.6 [(a), (b)]	1.8 ± 0.6 [(c), (d)]
Sucrose	2	16.6 ± 2.2	1.8 ± 0.5
BSA	0.2	12.1 ± 4.8	2.5 ± 1.3
	2	5.5 ± 5.5 [(a)]	3.6 ± 1.5 [(c)]
Fetuin	0.2	8.2 ± 7.1 [(b)]	3.5 ± 1.4 [(d)]
Histone H1	0.01	15.4 ± 1.7	1.7 ± 0.8

Notes: *p*-values are <0.001 (a), <0.001 (b), <0.005 (c), and <0.005 (d).
